# Host genetics and the rumen microbiome jointly associate with methane emissions in dairy cows

**DOI:** 10.1371/journal.pgen.1007580

**Published:** 2018-10-12

**Authors:** Gareth Frank Difford, Damian Rafal Plichta, Peter Løvendahl, Jan Lassen, Samantha Joan Noel, Ole Højberg, André-Denis G. Wright, Zhigang Zhu, Lise Kristensen, Henrik Bjørn Nielsen, Bernt Guldbrandtsen, Goutam Sahana

**Affiliations:** 1 Center for Quantitative Genetics and Genomics, Department of Molecular Biology and Genetics, Aarhus University, Tjele, Denmark; 2 Wageningen University & Research, Animal Breeding & Genomics, AH Wageningen, Netherlands; 3 Center for Biological Sequence Analysis, Dept. of Systems Biology, Technical University of Denmark, Kongens Lyngby, Denmark; 4 Clinical-Microbiomics A/S, Copenhagen, Denmark; 5 Viking Genetics, Randers SØ, Denmark; 6 Department of Animal Science, Aarhus University, Tjele, Denmark; 7 School of Animal and Comparative Biomedical Sciences, University of Arizona, Tucson, AZ, United States of America; University of Bern, SWITZERLAND

## Abstract

Cattle and other ruminants produce large quantities of methane (~110 million metric tonnes per annum), which is a potent greenhouse gas affecting global climate change. Methane (CH_4_) is a natural by-product of gastro-enteric microbial fermentation of feedstuffs in the rumen and contributes to 6% of total CH_4_ emissions from anthropogenic-related sources. The extent to which the host genome and rumen microbiome influence CH_4_ emission is not yet well known. This study confirms individual variation in CH_4_ production was influenced by individual host (cow) genotype, as well as the host’s rumen microbiome composition. Abundance of a small proportion of bacteria and archaea taxa were influenced to a limited extent by the host’s genotype and certain taxa were associated with CH_4_ emissions. However, the cumulative effect of all bacteria and archaea on CH_4_ production was 13%, the host genetics (heritability) was 21% and the two are largely independent. This study demonstrates variation in CH_4_ emission is likely not modulated through cow genetic effects on the rumen microbiome. Therefore, the rumen microbiome and cow genome could be targeted independently, by breeding low methane-emitting cows and in parallel, by investigating possible strategies that target changes in the rumen microbiome to reduce CH_4_ emissions in the cattle industry.

## Author summary

Methane is a potent greenhouse gas and ruminant livestock contribute a substantial amount of total methane from human activities. Variation between cows’ methane production has been found partly due to their genetics (heritable), making genetic selection a promising strategy for breeding low methane emitting cows. We hypothesized that the total methane production by a cow is affected by rumen microbes which are directly responsible for production of methane, as well as the cows’ own genetics and their interaction. We sampled the rumen contents of 750 dairy cows and found the relative abundance of some bacteria and archaea to be heritable and associated with methane production, but the majority of variation in relative abundance of rumen bacteria and archaea is due to non-genetic factors. We compared the amount of variation in methane production associated with host genetics as well as rumen bacteria and archaea and found the host genetics to explain 21% and rumen microbes 13%. Importantly, the two were largely independent of each other, so breeding for low methane emitting cows is unlikely to result in unfavorable changes in the rumen microbiome. However, further functional annotation of rumen microbiota is needed to confirm this. Strategies that target each source of variation can be conducted in parallel to optimize reduction in methane production from dairy cows.

## Introduction

Methane (CH_4_) is a potent greenhouse gas (GHG) with a climate change potential ~32 times greater than carbon dioxide (CO_2_)[1] and an atmospheric half-life of 12 years, which is substantially shorter than CO_2_ (> 100 years)[2]. Therefore, reducing CH_4_ emissions from anthropogenic-related sources has been identified as a key area for mitigating climate change with immediate effects[2,3]. Livestock accounts for 14.5% of anthropogenic-related GHG emissions and enteric CH_4_ emissions from ruminants accounts for 5.8%[3]. Furthermore, CH_4_ emissions from livestock is predicted to markedly increase due to an expected doubling in the global milk and meat demand by 2050[4].

Ruminants, the most widespread livestock species, can digest a wide variety of high fiber feedstuffs due to the distinct microbiome in their rumen. Methane is a natural by-product of gastro-enteric fermentation of high fiber plant biomass by microbial enzymatic activity in the rumen [5]. Bacteria, protozoa, and fungi in the rumen produce CO_2_ and hydrogen (H_2_), which are converted to CH_4_, primarily by archaea known as methanogens. Approximately 99% of CH_4_ emitted from cattle is released in the breath by eructation and respiration[6]. The emission of CH_4_ is also a crucial pathway for maintaining H_2_ balance and ruminal pH, as the optimal conditions for anaerobic fermentation by the rumen microbial community is limited to a narrow range of partial pressure of H_2_ and pH [7]. Hydrogenase-expressing bacteria convert metabolic hydrogen from anaerobic fermentation into H_2_ which is then converted to CH_4_ via methanogenesis [7]. Furthermore, emitted CH_4_ has a caloric value and represents a 2–12% net loss of a cow’s gross energy intake[8,9]. Consequently, cattle and other ruminants with increased efficiency to digest high fiber feedstuffs but reduced CH_4_ production could in principal benefit the global climate and concurrently improve the profitability and sustainability of cattle production.

Mitigation to decrease CH_4_ production by cattle to date has been largely unsuccessful, as the available measures are temporary and not cumulative. Large international research approaches target the rumen microbial communities through feed additives (chemical or biological), feed formulations, and anti-methanogen vaccines[10]. However, rumen microbial species rapid adaptation to changes in the substrate results in resistance to treatments and CH_4_ production returns to pre-treatment levels[11]. Conversely, rumen transplantation studies (transfaunation) show that the rumen bacterial community recovered to near pre-transfaunation composition after a short period of time[12]. This indicated the existence of a degree of host influence on rumen microbial composition[12]. Host genotype in cattle was reported to explain inter-animal differences in CH_4_ production[13,14] and the rumen microbial community influenced CH_4_ production[15]. However, empirical evidence linking the host’s genetic influence over the rumen microbial community and CH_4_ production is rather limited[15].

A promising strategy is genetic selection for low CH_4_ emitting cows, as it is sustainable, persistent, and cumulative over subsequent generations. Whether the host influences the rumen microbial community, and consequently CH_4_ production, or the two interact to affect CH_4_ production is currently unknown. If reduced CH_4_ production in cows is a consequence of poor symbiosis with rumen microbes and thus fiber digestibility, there is a risk selection for reduced CH_4_ production will act against the very symbiosis which has aided ruminants and rumen microbes’ coexistence. Thus, the extent to which the rumen microbiome is under the host genetic influence needs elucidation. If host genetics impose a strong influence on rumen microbial composition, traits influenced by rumen microbes could be improved by using rumen microbial composition as indicator traits in selection. However, should host genetics impose a strong influence on rumen microbial composition and selection for CH_4_ production proceed without cognizance of rumen microbial composition, there is a risk of unfavorable correlated responses in rumen microbial composition.

We hypothesized that: 1) the relative composition of the microbiome in the rumen is heritable i.e. controlled by host genome and 2) variation in methane emission from rumen is influenced by both the cow genome and rumen microbial content.

## Results

### Variation in methane emission and its heritability in lactating dairy cattle

Methane concentration in the exhalation-breath of 750 lactating Holstein dairy cows from farmer herds in Denmark was measured individually during automated machine milking for one week. Within-week methane measurements had a high repeatability coefficient of 0.70 ± 0.02 (estimate ± SE). Estimated average daily methane emission was 395.8 ± 63.5 g/d (mean ± SD), which was consistent with reports from the literature[16]. Considerable variation in estimated CH_4_ emission among cows was observed. The top 10% methane emitting cows (519.28 ± 28.5 g/d) had a 41% mean difference from the low 10% emitting cows (303.8 ± 11.9 g/d) (S1 Fig). Results from linear mixed model with pedigree records indicated methane emission was moderately heritable, 0.19 ± 0.09 (heritability coefficient, h^2^ ± S.E), which was consistent with previous findings in lactating Holstein cows in Denmark[13].

### Rumen bacterial and archaeal community composition

We identified 3,894 bacterial operational taxonomic units (OTUs, ≥ 97% identity) and 189 archaeal OTUs, which were present in a minimum of 50% of the cow samples (50% threshold maximizes the variation in a binary trait i.e. presence or absence). Taxonomic classification revealed generic bacterial and archaeal composition. The predominant bacterial phylum found was *Bacteroidetes* 72.2% ± 6.5 (mean ± SD), followed by *Firmicutes* (18.3% ± 5.6) and *Tenericutes* (2.8% ± 1.0). *Absconditabacteria*, *Spirochaetes*, *Fibrobacteres*, and *Proteobacteria* each comprised less than 2%, and another 20 phyla constituted 1% of all sequence reads.

The archaeal community was dominated by two families, *Methanobacteriaceae* and *Methanomassiliicoccaceae* (35% ± 22.1) of the orders *Methanobacteriales* (64.2% ± 22.2; mean ± SD) and the recently proposed order *Methanomassiliicoccales* and class *Thermoplasmata*[17], respectively. The remaining archaeal community was comprised of 10 families, which were low in abundance, cumulatively accounting for less than 1% of all archaeal sequence reads.

### Additive genetic variance estimates of rumen microbiota

**Fig 1:**
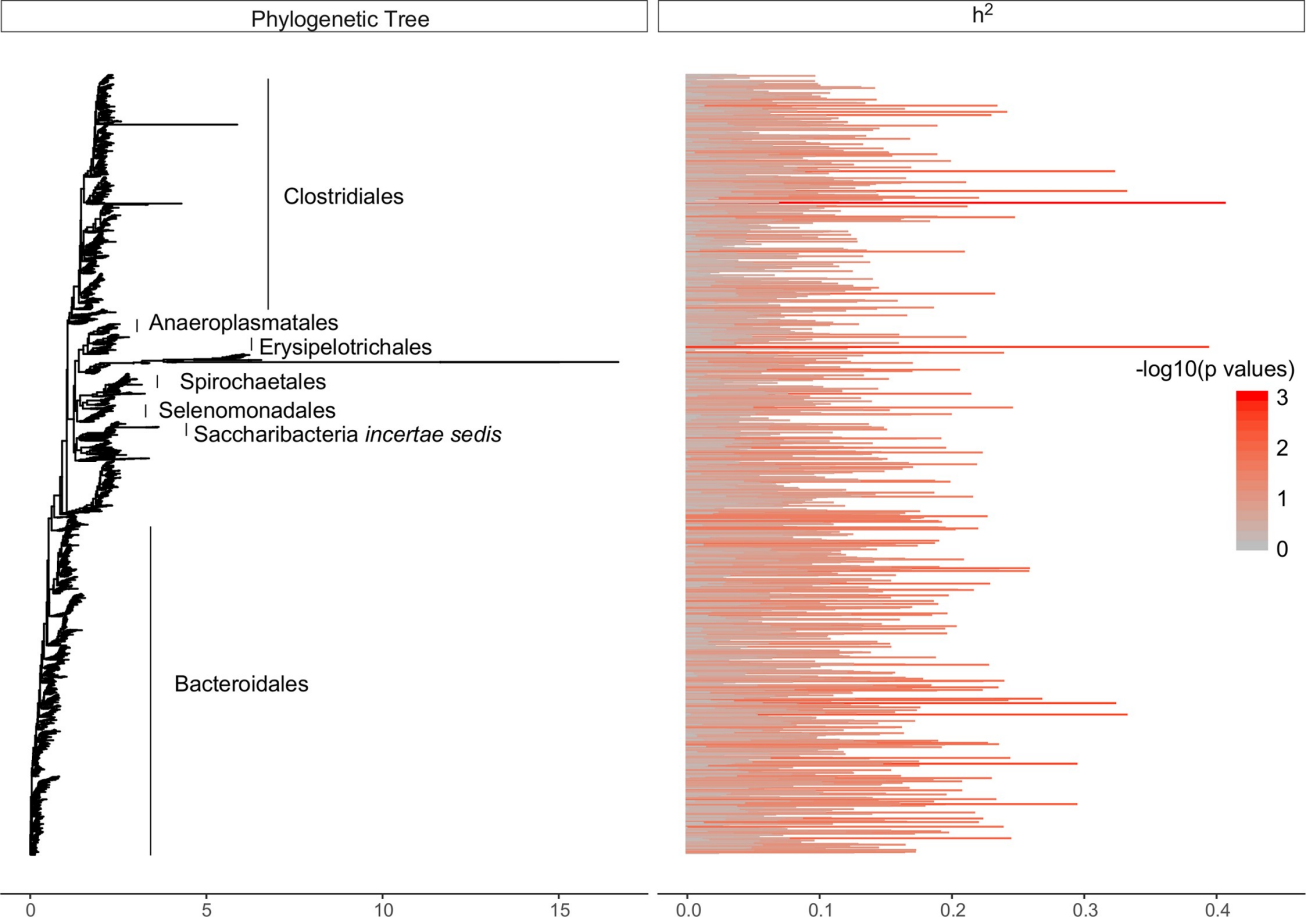
Phylogeny of 3,894 rumen bacterial OTU. Branch lengths represent substitution number per site calculated by FastTree2[81]. Heritability estimates (h^2^) for each OTU abundance are plotted with a horizontal bar and colored by taxonomic group classification.

**Fig 2:**
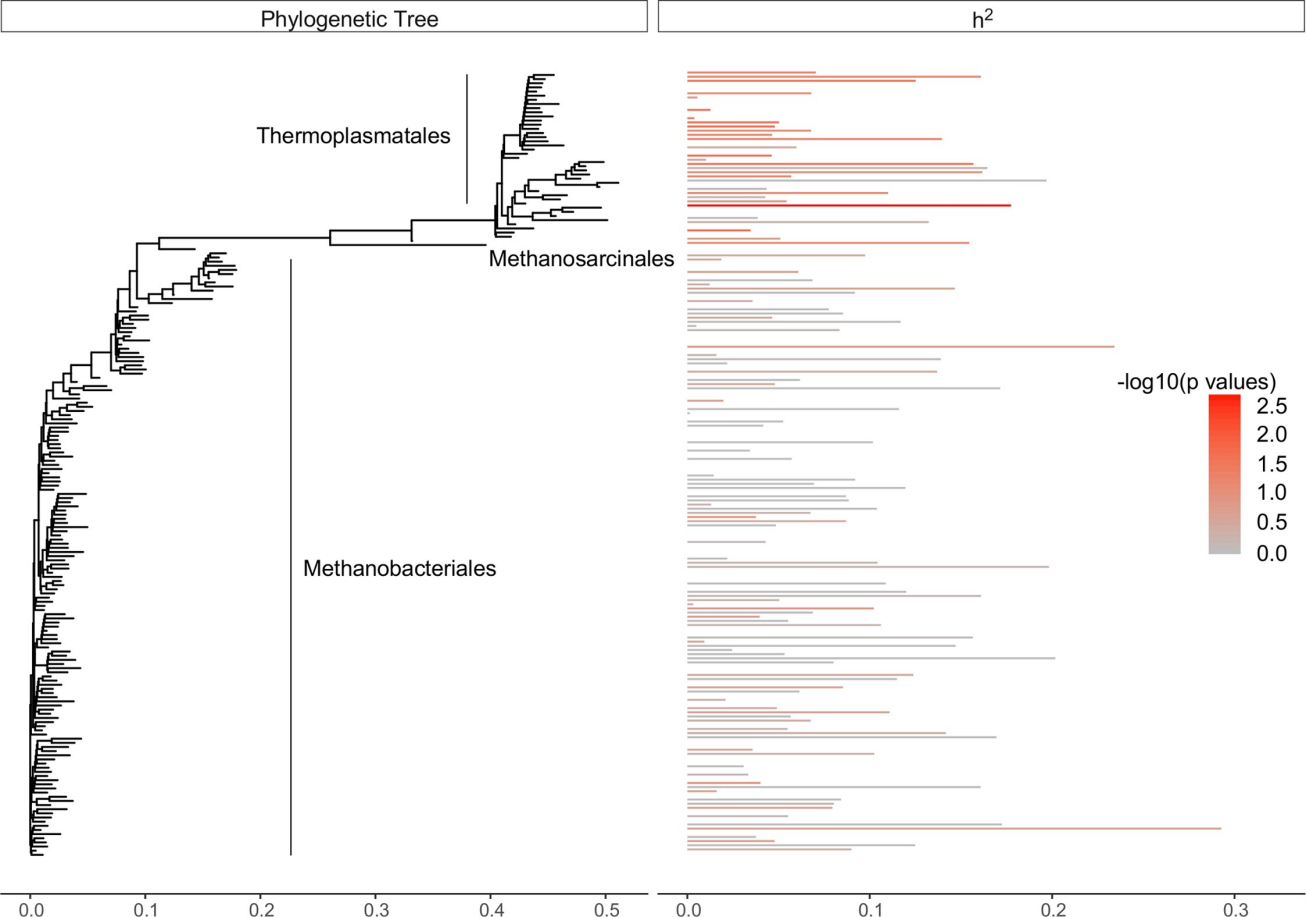
Phylogeny of 189 rumen archaeal OTU abundance. Branch lengths represent substitution number per site calculated by FastTree2[81]. Heritability estimates (h^2^) for each OTU abundance is plotted with a horizontal bar and colored by taxonomic group classification.

OTU abundance and OTU abundance collapsed at genus and family levels were used as microbial phenotypes. The heritability thereof was estimated using a linear mixed model with pedigree records (known as ‘animal models’), which partitions total variance into additive genetic and environmental variance[18]. We calculated 95% confidence intervals for OTU h^2^ estimates and found for 6% of bacterial and 12% of archaeal OTUs, the estimates were significantly higher than zero (*P* < 0.05), ranging from 16–44% (Fig 1) and 18–33% (Fig 2), respectively. Due to the high number of independent tests, we calculated false discovery rate (FDR) corrected *P*—values for h^2^ estimates with a FDR threshold of 15% (S1 Table).

Heritability of bacterial and archaeal abundance was further estimated at the genus level. In total eight bacterial genera out of 144 showed significant h^2^ estimates ranging from 0.17 to 0.25 (Table 1). Only a single archaeal genus, *Methanobrevibacter*, had a h^2^ estimate significantly different from zero (0.22 ± 0.09). However, *Methanosphaera* and *Methanomicrococcus* might also be under host additive genetic control with heritability estimates approaching significance thresholds (Table 1).

**Table 1:** Estimated heritability (h^2^) and P-value for the relative abundances of bacterial and archaeal genera.

Genus	Relative abundance (%)	h^2^± SE	95% Confidence Interval	*P*-value
**Bacteria**				
*Paludibacter*	0.01	0.25 ± 0.10	(0.05–0.45)	0.015
Unclassified *Spirochaetaceae*	0.01	0.25 ± 0.09	(0.04–0.41)	0.08
*R4-45b*	0.01	0.23 ± 0.09	(0.05–0.41)	0.014
*F16*	0.8	0.22 ± 0.09	(0.04–0.40)	0.018
Unclassified *Endomicrobia*	0.04	0.21 ± 0.09	(0.02–0.40)	0.027
Unclassified *Victivallaceae*	0.08	0.20 ± 0.09	(0.01–0.39)	0.36
Unclassified *Proteobacteria*	0.02	0.19 ± 0.09	(0.01–0.37)	0.042
*Sporobacter*	0.01	0.17 ± 0.08	(0.00–0.34)	0.046
**Archaea**				
*Methanobrevibacter*	55.8	0.22 ± 0.09	(0.04–0.42)	0.02
*Methanosphaera*	8.1	0.18 ± 0.10	(-0.00–0.36)	0.055
*Methanomicrococcus*	0.7	0.18 ± 0.09	(-0.02–0.38)	0.08

### Association between microbiota abundance and methane production

**Fig 3:**
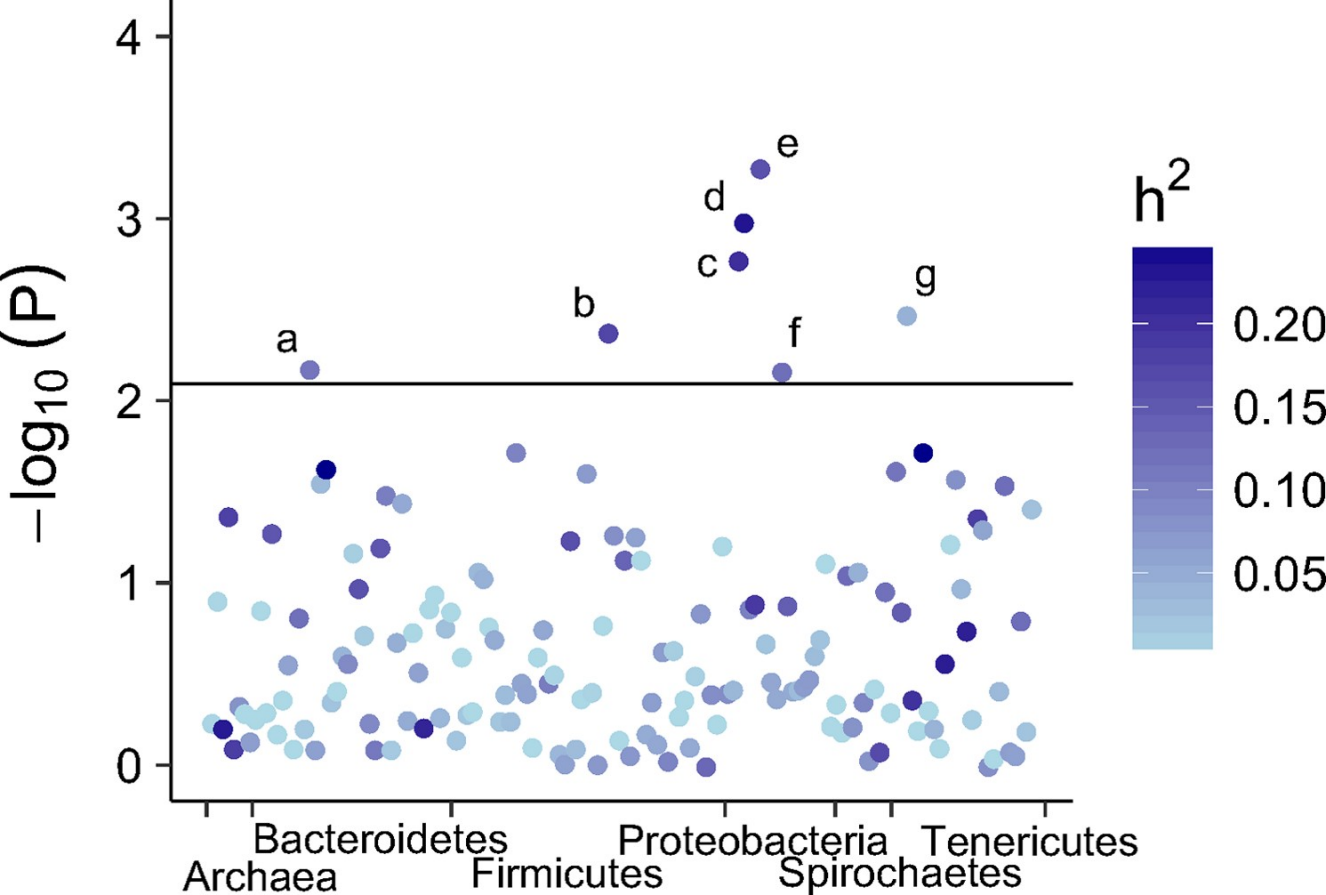
Manhattan plot of rumen bacterial and archaeal genera associations with methane emissions (g/day) colored by heritability (h^2^) estimates. Color gradient indicates genera h^2^ with light blue (h^2^ = 0) ranging to dark blue (h^2^ = 0.30). The y-axis is -log_10_(*P*) for association tests. The horizontal line represents the Benjamini-Hochberg FDR 15% for multiple testing significance thresholds. Genera above the threshold are a) Unclassified *BS11* group; b) *Sporobacter*; c) Unclassified *Victivallaceae*; d*)* Unclassified *Lentisphaeria*; e) Unclassified *Alphaproteobacteria*; f) Unclassified *Rickettsiales*; and g) *Sphaerochaeta*.

Associations between relative bacterial and archaeal OTUs, genera abundance, and host CH_4_ emissions were tested, while simultaneously controlling for environmental factors and familial structures common in livestock due to relatedness among study samples [19,20]. The OTU or genera log-transformed abundance present in > 50% of cows were fit as an explanatory variable in a linear mixed model for CH_4_ production. Numerous significant OTUs were detected but failed to pass the threshold for multiple testing (FDR ≤ 0.15) (Supplementary Table 1). This was a hypothesis-generating analysis and not directed at specific hypothesis testing therefore we reported the significance and FDR corrected values (S1 Table). Seven genera in total were detected, which exceeded the significance threshold at FDR of 15%. The -log_10_ *P*-values are plotted in Fig 3.

### Microbial community structure

**Fig 4:**
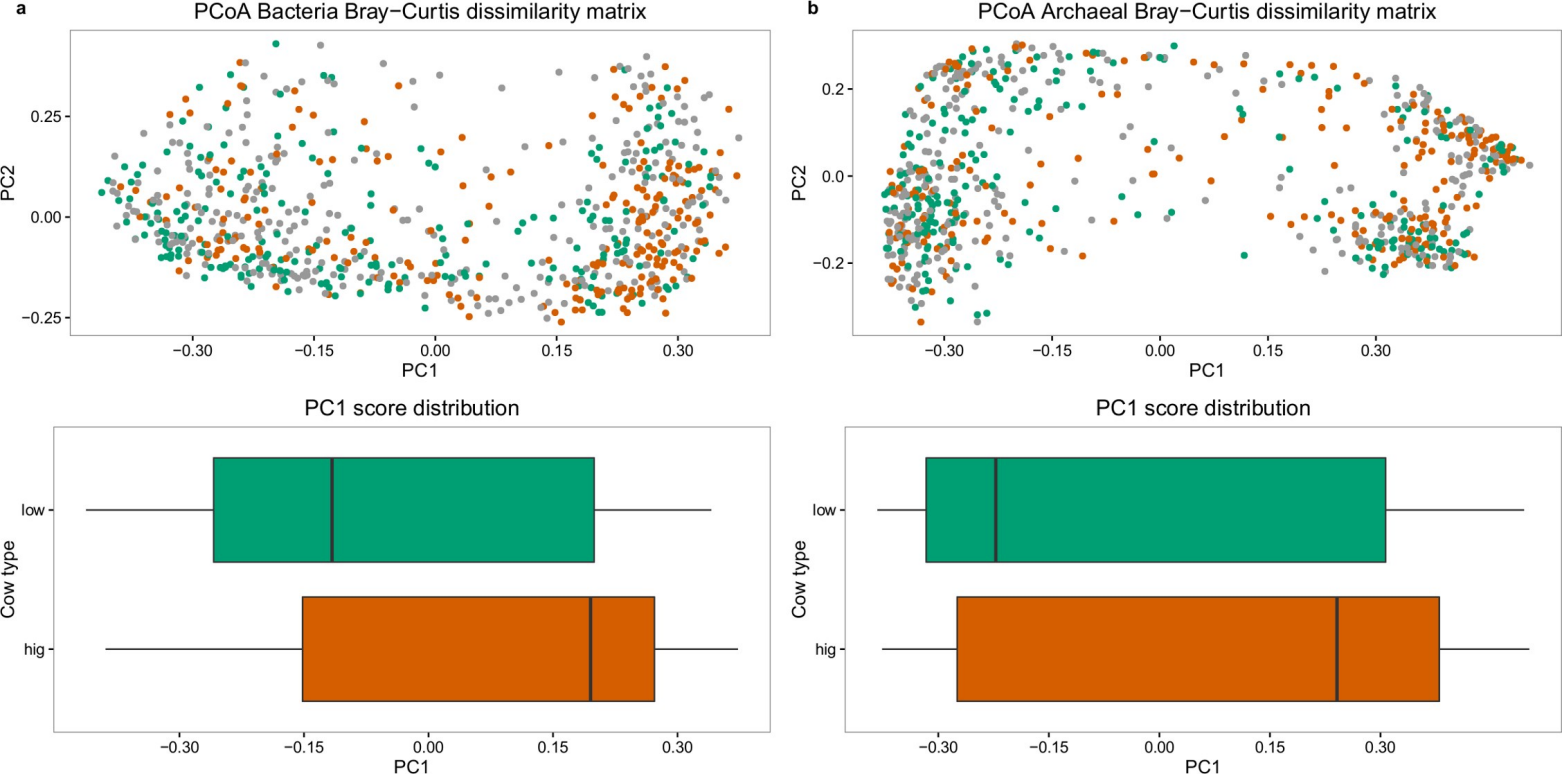
Principal coordinate analysis (PCoA) of rumen bacterial community diversity (a) and archaeal community diversity (b) based on 16S rRNA amplicon sequencing contrasting 10% highest methane emitters (orange), 10% lowest methane emitters (blue), and 80% intermediate emitters (grey). Distribution of high and low emitters along PCo1 showed significant differences (*P* < 0.001) for both figures.

Traditionally, dimension reduction techniques such as principal coordinates analysis (PCoA) are used to summarize community composition differences between individuals (Beta diversity) into clusters, which are further examined for associated biological or explanatory variables. Differences in bacterial and archaeal community structures were estimated for the entire sample population at OTU level using the Bray-Curtis[21] dissimilarity metric (PCoA, Fig 4A and 4B). Briefly, the Bray-Curtis dissimilarity is the sum of minimum counts of shared species in two animals divided by the sum of counts of all species in each animal, where 0 indicates the same composition and 1 indicates no shared composition. Analysis revealed clustering of cows into ‘ruminotypes’ for both bacterial and archaeal community composition, which both associated significantly with high and low CH_4_ emitters at opposing polar regions (Mann-Whitney test, *P* < 0.001) but failed to cluster distinctly from the intermediate CH_4_ emitters. Analysis of community structures using ANOVA revealed bacterial PCo1 was partly explained by non-genetic factors: parity (i.e. lactation number) (3.6%), sequencing batch (2%) and lactation stage (1%). A genetic analysis controlling for these factors showed PCo1 was likely heritable (0.20 ± 0.10) and thus influenced by the host additive genetics. Bacterial PCo2 was partly explained by the herd of origin (< 1%) and parity (< 1%) and was not heritable (0.02 ± 0.05). Similar findings were observed for archaea, with the variation in PCo1 partly explained by herd (< 1%), parity (19.9%), sequencing batch (5%) and lactation stage (< 1%). The genetic analysis controlling for these factors exhibited moderate heritability (0.39 ± 0.05). Archaeal PCo2 variation was partly explained by herd (< 1%) and parity (< 1%), which were likely not heritable (0.05 ± 0.05).

### Variation in methane emission attributed to cows’ additive genetics and rumen microbiome

**Fig 5:**
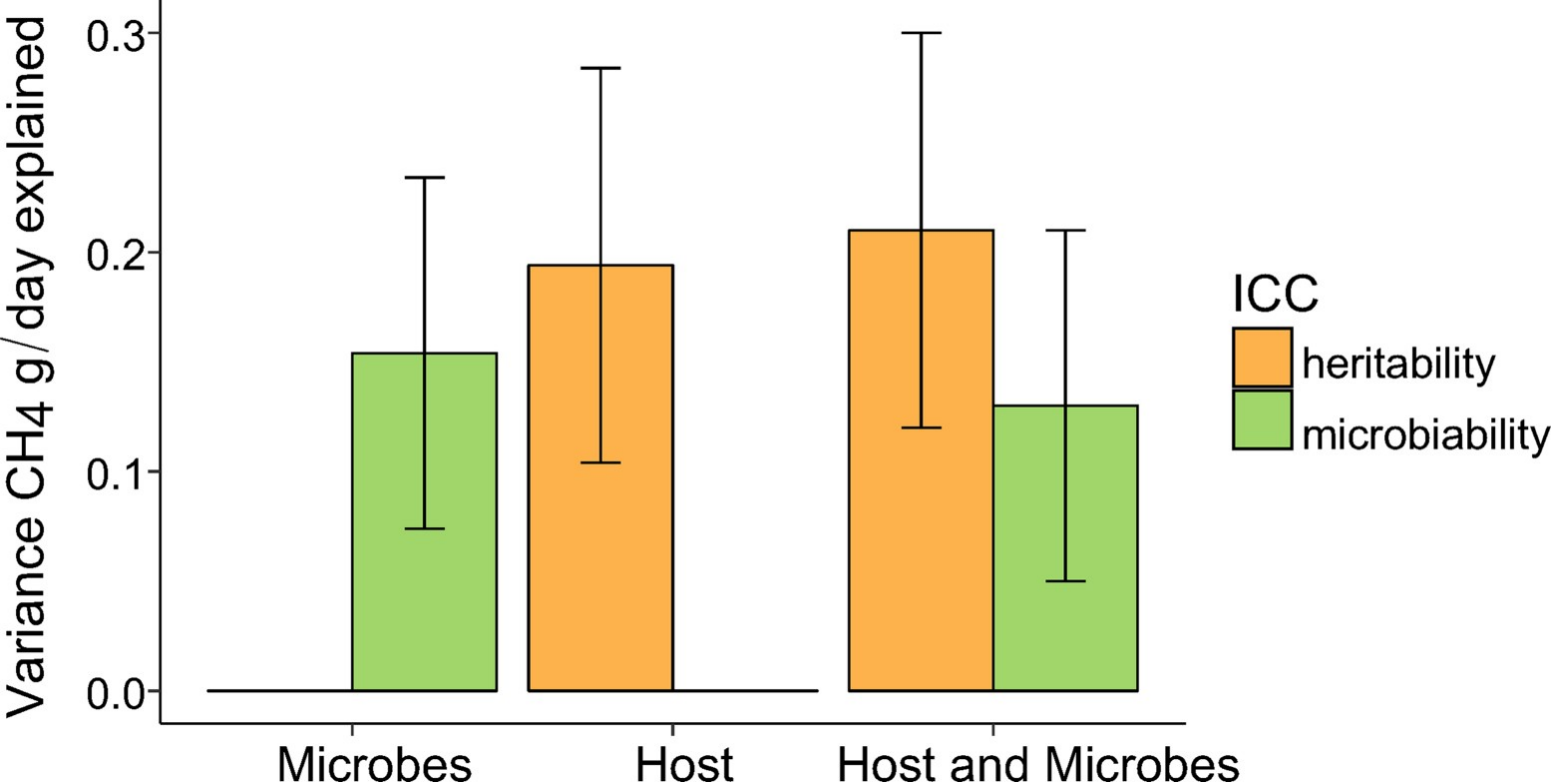
Proportion of variance in CH4 explained by different sources (Intra-class correlation coefficients, ICC) due to additive genetic effects (heritability) and rumen microbe content (microbiability), with respective standard errors when fit separately or jointly.

The relative proportion of variation in CH_4_ emissions due to rumen microbial composition and host additive genetic components was estimated individually and jointly using linear mixed models. Likelihood ratio tests revealed that fitting either random effect of rumen microbial composition or individual cow’s polygenic component fitted the data significantly better than the null model i.e. including only fixed effects (*P* < 0.001). The model fitting both random effects (microbial composition and polygenic component) was significantly better (*P* < 0.001) than models including only one random effect. The proportion of variance in CH_4_ production explained by the microbiome, here defined as microbiability (m^2^), was calculated in analogy to the heritability (h^2^)[22,23]. The contrast between the two intra-class correlation coefficients h^2^ and m^2^ with their respective standard errors for all models are depicted in Fig 5. The m^2^ of CH_4_ emission estimated individually was 0.15 ± 0.08 (estimate ± S.E) and the h^2^ estimated individually was 0.19 ± 0.09. Simultaneous estimates of both effects indicated slightly lower microbiability (0.13 ± 0.08), whereas h^2^ exhibited a corresponding increase (0.21 ± 0.09) as compared to the preceding models fitting only one of the random effects. The combined microbial abundance and additive genetic effects were responsible for ~ 34% of the total phenotypic variation in CH_4_ emissions.

## Discussion

### Additive genetic variation in methane emissions and bacterial and archaeal taxa abundance

The results of this study show that estimated CH_4_ emissions from a dairy cow were partially under the influence of host (cow’s) additive genetics, which explained 19% of the total variation. Of the rumen bacterial OTUs, a modest ~ 6% were associated with host additive genetics exhibiting significant heritability estimates (16–44%) (Fig 1). Similarly, only ~ 12% of archaeal OTU abundance was influenced by host additive genetics, with heritability estimates ranging from 18–33% (Fig 2). However, bacterial and archaeal heritability estimates failed to pass the threshold for multiple testing. Our test was conservative as a large number of taxa were analyzed with many OTUs having little or no influence by the host genome. Studies with larger sample sizes would give more reliable estimates of the heritabilities, especially for lower heritable OTUs. The h^2^ estimates observed in this study were consistent with findings of intestinal microbiota in mice[24,25] and humans[26,27] and confirm that the majority of variation in rumen microbial abundance is due to factors other than host additive genetics [28]. Interestingly, the patterns of h^2^ with phylogeny differed between the bacteria and the archaea (Fig 1 and Fig 2). Heritable OTUs were distributed throughout the bacterial microbiome whereas archaea showed increased heritability within the *Thermoplasmatales*. This highlights the value of collating phylogeny with heritability estimates to focus research into possible mechanisms which predispose differential relative abundance of certain taxa across genetically related cows. The method employed to sample rumen contents is high-throughput and less invasive than surgical procedures, making it better suited to sampling large numbers of cows under commercial farm conditions. Large sample size is critical in genetic evaluations. However, it is important to note that the floral rumen scoop is inserted into an undefined portion of the rumen and likely samples the liquid phase. Recognizing that rumen microbial communities differ between liquid, solid and epimural phases[29], studies testing the repeatability and representativeness of sampling are needed.

### Associating rumen microbial taxa abundance with CH_4_ emissions

We utilized linear mixed model analysis to test for associations between bacterial and archaeal OTUs, genera and families with estimated CH_4_ emissions, while concurrently accounting for effects such as parity, lactation stage, herd of origin and familial structure from the pedigree. Several bacterial genera associated with CH_4_ emission were detected. Out of these, four were found either to be affected by methane inhibitors or related to H_2_ production and other methanogenesis substrates. Three were moderately heritable (0.17–0.25) (S1 Table). One of the identified bacteria, *Sporobacter*, with a mean relative abundance of 0.01% (*Ruminococcaceae*, *Clostridiales*, *Firmicutes*), belongs to a group with only a single cultured representative, *Sporobacter termitidis*, isolated from the intestine of wood-feeding termites (*Nasutitemes lujae*), also known for producing large amounts of CH_4_. However, when this isolate was co-cultured with an archaea species, *Methanospirillum hungatei*, CH_4_ was not produced. *S*. *termitidis* was found to generate acetate and methylsulfides, but not H_2_ or CO_2_, therefore interspecies H_2_ transfer did not occur and facilitate CH_4_ production[30]. The recent discovery and proposed archaeal order *Methanomassiliicoccales* species found to utilize methylsulfides and H_2_ in methanogenesis[31], provides a possible mechanism for methylsulfide producers to contribute to CH_4_ production when H_2_ producers are present. *Methanomassilicoccales* was prevalent in our samples (mean relative abundance 35%); therefore, *Sporobacter* could potentially be contributing to CH_4_ production via a similar pathway.

We also detected *Sphaerochaeta* with a mean relative abundance of 0.01%, associated with estimated CH_4_ production. Genomes from cultured *Sphaerochaeta* isolates revealed acetate, formate, ethanol, H_2_, and CO_2_ were potential fermentation end products[32], many of which are methanogenic archaea substrates[33]. Furthermore, seed extracts from *Perilla frutescens* (*Lamiaceae*), a medicinal herb, decreased CH_4_ production *in vitro* from rumen samples of lactating dairy cows and decreased *Sphaerochaeta* abundance[34]. Interestingly, Caro-Quintaro et al.[32] reported up to 40% of the genes from *Spaerochaeta* species were exchanged with members of *Clostridiales* (*Firmicutes*) and this inter-order-species horizontal gene transfer was most extensive in mesophilic anaerobic bacteria, such as the conditions found in termite and ruminant guts[35]. Here 16S rRNA gene sequencing is used as a proxy for metabolic activity but cannot account for inter-order-species horizontal gene transfer. Therefore, full metagenome sequence may have an advantage over the 16S rRNA gene to describe rumen microbial contents.

One bacterial genus detected in the present study, which is positively associated with estimated CH_4_ production, is classified in the yet uncultured BS11 gut group of the *Bacteroidales* (mean relative abundance 1.4%). The relative abundance of the BS11 group reportedly decreased concomitantly with CH_4_ production by dietary methanogenic inhibitors, such as *P*. *frutescens* seed extract, mentioned previously[34], monesin and essential oil supplementation in dairy cattle[36,37], and bromochloromethane in Japanese goats[38]. Thus, supporting our finding of a positive association between BS11 and CH_4_ production. Solden et al.[39] employed metagenomics sequencing and shotgun proteomics approaches to phylogenetically and metabolically resolve the BS11 gut group. They resolved two genera within the group and both exhibited multiple pathways to ferment hemicellulose, a capability previously unknown for BS11. The resulting fermentation end products included acetate, butyrate, propionate, CO_2_, H_2_[39] the latter two being methanogenesis substrates. Genes encoding ‘fucose sensing’ pathways were found for only one of the proposed BS11 genera, offering a possible mechanism for interaction between genes in the BS11 group and the host[15]. However, further studies are needed to elucidate the links between CH_4_ inhibitors, host genes and CH_4_ production.

Due to the absence of cultured rumen bacteria isolates, an understanding of the metabolic function in many bacterial genera remains in its infancy. However, from the isolates discussed above, results suggested CH_4_ emissions depend on abundance of bacterial taxa that produce substrates for methanogenesis, such as H_2_. Remarkably, associations between archaeal relative abundance and estimated CH_4_ production were not detected in the present study, despite the knowledge that archaea are directly responsible for CH_4_ production. A meta-transcriptome study in sheep found archaeal transcription pathways and not simply abundance, contributed to inter-animal differences in CH_4_ production[40]. This study was congruent with conclusions reached in two recent reviews, which examined results from dairy cattle and other ruminant studies employing 16S rRNA[41] and ‘meta-omics’ approaches[42], where bacteria abundance produced and utilized H_2_ or stabilized pH, which affected CH_4_ emissions and feed efficiency and archaeal activity matched substrate availability.

### Associating rumen community structure with methane emissions

The combined effects of the bacterial and archaeal community structure (beta diversity) on estimated CH_4_ emissions were investigated by conducting PCoA on the archaeal and bacterial communities, which revealed 2–3 clusters for archaea (Fig 4A) and two clusters for bacteria (Fig 4B). Beta diversity is a non-parametric distance measure used in microbiology and ecology to assess the differences between environments or samples (in this case cows) as opposed to alpha diversity which takes into account the diversity within cows. Clusters of a similar nature were first reported in intestinal bacterial community types in humans[43,44], chimpanzees[45], mice[46] and pigs[47], referred to as “enterotypes”, and found associated with specific host phenotypes. This concept was extended to sheep rumen bacterial communities and referred to as “ruminotypes”[48]. The ruminotypes observed herein followed a continuous gradient and did not form discrete clusters, which is consistent with the latest findings in microbiome stratification. [49]. Importantly, we found that animal and farm factors like herd of origin, parity and lactation stage, as well as technical factors, i.e. sequencing batch, contributed to the observed variation and stratification in ruminotypes. Similar findings were reported in rumen bacterial richness at different lactation stages and over different parities[50], suggesting later parities (higher parity cows are older) decreased bacterial richness and increased production[51]. We detected a moderate heritable genetic component acting along PCo1 axis, with h^2^ of 20% for bacterial and 39% for archaea, when controlling for lactation stage and parity, demonstrating the first evidence of host additive genetic influence on rumen bacterial and archaeal community structure (beta diversity). All the above-mentioned factors contribute to microbiome structure and associations with host phenotypes.

An association was detected between the highest and lowest CH_4_ emitters and bacterial and archaeal ruminotypes along PCo1, however, ruminotype cluster memberships were not exclusive to high and low emitters. This suggested ruminal bacterial and archaeal community structure provided a modest contribution to CH_4_ emission. Kittlemann et al.[48] surveyed microbial community composition in multiple sheep cohorts with low and high CH_4_ yield (methane emission per kg dry matter intake, CH4/DMI). A ruminotype “S” associated with low CH_4_ yield and enriched with *Sharpea azabuensis* was reported. A follow up study in sheep also found low CH_4_ yielding sheep to be associated with ruminotype “S”, enriched with *Sharpea* spp. It was hypothesized a smaller rumen size and higher turnover rate promoted faster growing bacteria, such as *Sharpea*, which favor hetero-fermentative growth on soluble sugars, resulting in lower H_2_ production and subsequently decreased CH_4_ formation by hydrogenotrophic methanogens[52]. Smuts et al.[53] reported passage rate (and consequently turnover rate) in sheep was heritable, indicating a possible mechanism for host genetics to influence ruminotypes. Methane emission phenotypes differed between the sheep and the present study. Kittlemann et al.[48] assessed the amount of CH_4_ production per unit of DMI but not CH_4_ production directly. DMI measurements are not currently recorded on dairy cattle under commercial farms due to the high costs and therefore, CH_4_ emissions in the present study could not be corrected for feed intake. In light of the differences in phenotype definitions and similarities in ruminotypes between studies, it would be of interest in future work to obtain DMI records on cows and test if the ruminotypes observed show an increased relationship with CH_4_ yield. The heritability estimates for PCo1 and PCo2 indicates these measures could potentially be used as indicator traits in genetic selection should they be highly correlated to a trait of interest, however PCo1 and PCo2 (beta diversity) does not account for the total rumen microbial variation within and between individuals.

The method employed to measure CH_4_ production in the present study is high throughput and non-invasive, making it practically viable for measuring large numbers of animals under commercial farm conditions. However, the cost trade off of this method is that it makes use of milk yield and body weight in the estimation of CH_4_ production. Validation of this method with the ‘gold standard method’ (climate respiration chambers) has yielded highly correlated (r = 0.8–0.89) and concordant (concordance correlation coefficient = 0.84) results in dairy cattle [54,55]. However, the effects of body weight and milk yield on estimation of CH_4_ cannot be discounted and further research into the relationships between these variables and the rumen microbiome would be of value.

### Quantifying variation in CH_4_ due to cow additive genetic effects and rumen microbiome

In this study, we quantified the combined effects of all rumen bacterial and archaeal OTUs simultaneously on estimated host CH_4_ emissions using a microbial relationship matrix among cows. This is a parametric approach similar to assessing both alpha and beta diversity, as total rumen microbial variation within and between individuals is taken into account simultaneously. We expressed the combined effects as the variance ratio due to microbial composition to the total variance in estimated CH_4_ emissions (m^2^, microbiability), an analogy to h^2^. Estimated CH_4_ emissions had 15% m^2^, indicating the combined rumen bacteria and archaea abundance of dairy cattle was associated with a considerable amount of variation in estimated CH_4_ emissions among animals. Ross et al.[56] first proposed the generation of metagenomic relationship matrices in dairy cattle and reported a CH_4_ emission prediction accuracy of 0.47, explaining 22% of the total variation in CH_4_ production [57]. However, Ross et al. [57] did not have sufficient data to estimate h^2^ or microbiability (m^2^) in CH_4_ production. A study with 207 pigs employing 16S rRNA sequencing of gut microbes, found eight of the 49 bacterial genera to be heritable and estimated m^2^ and h^2^ for feed intake (m^2^ = 0.16, h^2^ = 0.11), daily gain (m^2^ = 0.28, h^2^ = 0.42) and feed conversion ratio (m^2^ = 0.21, h^2^ = 0.19) [23]. Only daily gain had higher h^2^ compared with m^2^. These findings suggest agreement with holobiont theory, where variation in the genome and microbiome can cause variation in some complex traits, on which artificial, natural selection and genetic drift can act [58,59]. However, the aforementioned study did not have adequate numbers of animals to estimate m^2^ and h^2^ simultaneously to assess the relative interactions between additive genetics and the microbiome. Thus, it was unable to assess if host additive genetics co-influences the microbiome and variation in phenotypes.

In contrast, we estimated m^2^ and h^2^ concurrently to examine the shared information between the two effects. Microbiability of estimated CH_4_ production decreased by two percentage points to 13% and h^2^ exhibited a corresponding increase from 19 to 21%. This result indicated host genetic effects do interact with the microbial community composition but are not the primary mechanism for host genetic effects on estimated CH_4_ emissions. A possible explanation for the negligible amount of shared influence between the two relationship matrices might be the small percentage of heritable bacterial and archaeal OTUs. This implies that the rumen bacterial and archaeal communities affected estimated host CH_4_ emissions independently and host genetics influenced a small portion of these bacteria and archaea. The combined host additive genetics and rumen microbial community composition explained ~ 34% of the total variance in estimated CH_4_ emissions in dairy cattle. Thus, breeding for low CH_4_ production can be expected to result in limited correlated genetic responses to shape the rumen microbiome and breeding can likely proceed without taking cognizance of the rumen microbiome for this trait. However, larger studies estimating genetic correlations between rumen microbiota and CH_4_ emissions and better functional annotation of rumen microbiota are needed to confirm this.

Microbiability estimates can be used as a tool for quantifying the cumulative effects of microbial abundance on phenotypes, e.g. complex diseases and quantitative traits. However, further research is required to elucidate the biological mechanisms shaping microbiability. For example, animal factors known to affect CH_4_ production and rumen microbial populations, such as passage rates or individual differences in feed intake might influence microbiability estimates. Human intestinal microbiome studies find that numerous disease phenotypes are associated with microbial richness, species abundance, and microbial community structure[60,61]. Subsequent work using stool consistency and opaque markers as proxies for colonic transit time found all three metrics and disease phenotypes are partially confounded with colonic transit time[62,63]. Similarly, in sheep studies, low CH_4_ yielding sheep are associated with lower retention time and smaller rumens[64], relationships with specific rumen microbial clusters[48] and different bacterial and archaeal species[52]. Therefore, studies are needed to determine if microbial differences among subjects associated with phenotypic differences are causative or are consequences of unknown extraneous factors. It is also necessary to clarify the mechanisms which allow rumen microbes to be passed on to successive generations, to assess the efficacy of perturbations of the rumen microbiome such as probiotics and rumen transplants aimed at desired changes to the rumen microbiome and associated changes in phenotypes[65]. Regardless of the underlying biology, quantifying the relative contribution of rumen microbes and additive genetics to complex phenotypes helps characterize whether the host genome and microbiome are acting jointly as a holobiont and highlights the merits of targeting microorganisms to achieve a specific change in a phenotype or selective breeding. Furthermore, providing additional information, such as relative abundance of rumen fungi and protozoa, or ‘meta-omics’, including meta-transcriptomics or meta-proteomics data can be readily adopted and incorporated into this methodology, offering insights into economically important livestock and disease traits in humans.

## Conclusions

Methane production by dairy cows is not only influenced by factors such as feed intake and composition among others, but also the cow’s individual genetic composition and rumen microbial composition. Each cow’s additive genetic effects influence a modest amount of variation in the abundance of a small percentage of rumen bacterial and archaeal taxa, and thereby contribute to variation in rumen microbiome composition and function. We detected associations between CH_4_ emissions and rumen bacteria abundance, which are known to produce methanogenesis substrates, suggesting bacteria driven CH_4_ production pathways. Although we detected a heritable component to ruminotypes, the association to CH_4_ production was weak. Concurrently, host additive genetic effects and rumen microbes contributed to inter-animal differences in CH_4_ production, however negligible interaction was observed between microbiability and heritability. Consequently, cow additive genetic effects on CH_4_ emissions were largely unmodulated by cow additive genetic effects on rumen bacteria and archaea abundance. Strategies to reduce CH_4_ emissions in ruminants can be optimized by a multifaceted approach, for instance, selective breeding to unlock host’s genetic potential and strategies which may effect desired changes in the rumen microbiota like rumen transplantation, and probiotics.

## Materials and methods

### Experimental design

Methane emissions from 750 lactating Holstein cows in five commercial herds were recorded using a portable Fourier Transform Infrared unit (FTIR; Gasmet DX-4000, Gasmet Technologies, Helsinki, Finland)[13,66] and one research herd using a permanently installed non-dispersive infrared (NDIR; Guardian NG/Gascard Edinburgh Instruments Ltd., Livingston, UK)[67]. Briefly, the FTIR and NDIR equipment were installed within the feed bins of automated milking systems (AMS) in each commercial herd with the FTIR for seven consecutive days and the NDIR were permanently placed in the research herd. The FTIR and NDIR device inlets were installed in the AMS feed bins and methane (CH_4_) and carbon dioxide (CO_2_) gas concentrations (ppm) sampled continuously every 5 s and 1 s, respectively[66,67]. Cows were milked individually in the AMS and milked on average (18.2 ± 3.4) times during the seven-day period, for durations ranging from five minutes to 12.2 minutes. Mean CH_4_ and CO_2_ gas concentrations were corrected for environmental factors, including diurnal variation and day to day differences using a linear mixed model following Difford et al.[67] to approximate daily averages. Measurement stability was assessed by model repeatability and used as data quality control. All herds practiced indoor feeding strategies with *ad libitum* access to feed and water. A total mixed ration (TMR) was provided, consisting primarily of rolled barley, corn silage, grass clover silage, rapeseed meal, soybean meal and up to 3 kg of concentrate supplement given during milking. Although all commercial herds employed a standardized TMR recipe, ingredient-specific differences among farms were expected to contribute to differences in TMR dietary values over herds.

Weekly mean values for milk yield and body weight were combined with weekly gas concentrations, as described in Lassen et al. [66] and applied to predict cow heat production[68]. During each week of CH_4_ and CO_2_ recording at different herds, milk samples were collected to estimate milk fat and protein percentages. Cow fat and protein corrected milk yield (FPCM) was estimated following the national recording scheme (RYK, Skejby, Denmark)[69]. Methane production (L/day) was estimated using the CH_4_ to CO_2_ ratio and predicted CO_2_ emission[70] from the conversion of cow heat production units to CO_2_ production, following Madsen et al.[71] and then converted to (g/d) using CH_4_ density at standard temperature and pressure.

Holstein cow pedigree records were traced in the Danish national database (NAV, Skejby, Denmark) as far back as 1926 to construct a pedigree-based relationship matrix for the quantitative genetic analysis.

### Sampling rumen liquid fraction

Immediately following the CH_4_ recording period, rumen content samples were drawn from individual cows by oral insertion of the probe “Flora Rumen Scoop” [72]. Approximately 40 mL of the liquid fraction containing particulate matter was drawn from the rumen using this method. Trained technicians conducted the sampling to ensure correct probe insertion into the rumen following a previously established protocol [72], recognizing that the location of the flora rumen scoop may differ somewhat from sampling to sampling. The entire “Flora Rumen Scoop” was rinsed vigorously between animal sampling to minimize cross-contamination. Samples were labeled, immediately placed on ice, and transferred to the laboratory within two hours for further processing. Each 40 mL sample was mixed vigorously, a subsample of 1.2 mL rumen fluid was collected, and transferred to a 1.5 mL vial, then snap frozen in liquid nitrogen, before storing at -80°C, until shipped on dry ice to a commercial sequencing company (GATC Biotech, Constance, Germany) for analysis.

### DNA extraction, bacterial and archaeal 16S rRNA gene amplification, and sequencing

DNA extraction, sequencing library construction and sequencing were conducted by GATC Biotech (Constance, Germany). Rumen samples were defrosted at 4°C overnight and vortexed until homogenous. A representative sample (500 μl) containing rumen liquid and solids was used for DNA isolation using the Qiagen QIAamp stool kit (Valencia, United States of America) following the manufacturer’s instructions, modified for the larger sample size[73].

Two primer sets were used to create 16S rRNA libraries, one set for all bacteria and one set for all archaea. Universal bacterial 16S rRNA gene primers (covering the V1-V3 variable regions) 27F: 5’-AGAGTTTGATCCTGGCTCAG-3’ and 534R: 5’-ATTACCGCGGCTGCTGG-3’ were used to generate the bacterial amplicon libraries (expected amplicon size 508 bp)[74]. Universal archaeal 16S rRNA gene primers (covering the V4-V6 variable regions) S-D-Arch-0519-a-S-15 5’-CAGCMGCCGCGGTAA-3’ and S-D-Arch-1041-a-A-18 5’-GGCCATGCACCWCCTCTC-3’ were used to generate the archaeal amplicon libraries (expected amplicon size 542 bp)[75]. Following protocols standardized by GATC Biotech, PCR amplifications were conducted with GoTaq Green polymerase (Promega, Madison, USA) with 30 PCR cycles and a 60°C annealing temperature for the archaeal amplicon libraries and 25 PCR cycles with a 60°C annealing temperature for the bacterial amplicon libraries. The 16S rRNA amplicons were purified using the Axyprep Fragment Select bead purification system (Axygen Biosciences, New York, USA), according to the manufacturer’s instructions. The size and purity of the PCR product was verified on a Fragment Analyzer using a High Sensitivity NGS Fragment Analysis Kit (Advanced Analytical Technologies, Ankeny, USA). Multiplex indices and Illumina overhang adapters were added to both amplicon libraries in a second PCR amplification round (six cycles), followed by Fragment Analyzer analysis to confirm the correct size of the amplicons (Advanced Analytical Technologies, Ankeny, USA). Ninety-six libraries were pooled in equimolar concentrations and sequenced with an Illumina sequencing instrument using the 300 bp paired-end read mode, according to the manufacturer’s specifications. Approximately half the samples were run using the illumina MiSeq platform and half with the HiSeq platform. The 300 bp paired end protocol was adapted to HiSeq by GATC Biotech. The specific samples entered into sequencing batches within each sequencing platform were recorded for subsequent significance testing to examine possible differences between sequencing batches and sequencing platforms in statistical analyses.

### Bacterial and archaeal 16S rRNA gene sequence processing and OTU table construction

Bacterial and archaeal sequence reads underwent quality control, processing and were clustered into operational taxonomic units (OTUs) using the LotuS pipeline[76] with the following options: Sequence truncation length and minimum sequence length after barcode and primer removal was 230 bp. Minimum average sequence quality score was 27, the maximum number of ambiguous bases was 0, maximum homonucleotide run was set to 8. Sequences were filtered away if any of the 50 bp segments in a sequence had average scores below 25 or if the expected number of errors exceeded 2.5 in the binomial error model. The low-quality sequence ends were trimmed by applying a sliding window quality filter with a width of 20 bp and a minimum average quality score within the window of 25. Sequences were truncated if the probabilistic accumulated error exceeded 0.75. The reads were de-replicated and sequences with a minimum of 10 replicates were retained for OTU clustering within the Lotus pipeline. Sequence pairs were merged with Flash[77] and clustered into OTUs based on sequence similarity (97%) with UPARSE[78] and chimeric sequences removed with UCHIME reference-based chimera detection[79]. Representative sequences from each OTU were aligned with ClustalO[80] and a phylogenetic tree built with FastTree2[81]. Representative sequences, the OTU table, and phylogenetic trees were transferred to QIIME (version 1.9.0)[82], where further analyses were performed. Taxonomy was assigned to each OTU using the RDP classifier with a confidence level of 0.8[83] using greengenes (gg_13_8_otus) as the reference database. Unclassified OTUs and OTUs classified to non-target kingdoms were filtered from the OTU tables, i.e. only OTUs classified as k_Bacteria were maintained for the bacterial primer set and similarly OTUs classified as k_Archaea maintained for the archaeal primer set. Finally, samples with < 50,000 sequences were removed and OTUs containing < 10 sequences were filtered out of the OTU table.

### Statistical models

#### Additive genetic variance estimation

The linear mixed model utilized to estimate additive genetic variance is as follows:

$$y_{ijkl}=\mu +h_{j}+p_{k}+b_{1}\left(dim_{l}\right)+b_{2}\left(e^{‑0.065\;x\;diml}\right)+a_{i}+e_{ijkl}$$ (1)

where y_*ijklm*_ is the observed phenotype, e.g. methane emission in grams/day; *μ* is the model intercept; *h*_*j*_ is the herd fixed effect (j = 6 levels); *p*_*k*_ is the parity fixed effect (k = 4 levels); *b*_*1*_ is days in milk fixed regression coefficient (*dim* l = 1–350); and *b*_*2*_ is the Wilmink term fixed regression coefficient generated on *dim* to account for non-linearity in early lactation [84]. Term *a*_*i*_ is individual animal random additive genetic effects ∼ NID(0, **A**σ^2^_a_), where σ^2^_a_ is the additive genetic variance and **A** is the pedigree derived numerator relationship matrix (i = 750 animals); and e_*ijkl*_ is the random residual ∼ NID(0, σ^2^_e_), where σ^2^_e_ is the error variance. The additive host genetic effects on relative rumen bacterial and archaeal abundance was estimated applying the same general equation as model 1 above, with the addition of the sequencing batch fixed effects nested within the sequencing platforms (11 levels). The analyses were performed using the DMU software[85].

#### Rumen microbial variance estimation

The relationship among cows based on their similarity in rumen microbiome composition was estimated by constructing a microbial relationship matrix (**M**) inspired by Ross et al.[56], where a metagenomic relationship matrix was created from a vector of aligned rumen microbial contig sequences. The matrix was computed as a variance-covariance matrix from rumen bacterial and archaeal abundance as follows:

$$M=\frac{XX^{\prime }}{n}$$ (2)

where **X** is the matrix of natural log transformed bacterial and archaeal relative abundance for all animals and n is the number of bacterial and archaeal OTUs within the population. Matrix **X** is derived from OTU tables after filtering out OTUs, which were absent from more than 50% of the samples and were homogeneous. The matrix **X** was subsequently scaled and centered within sequencing instrument (Miseq or Hiseq) to account for differences between instruments and recombined into a single matrix prior to the calculation of **M**.

The variance explained by microbial composition was estimated employing models similar to Eq 1, where the random effect of *m*_*i*_ was fit separately and jointly with *a*_*i*_, i.e. random additive genetic effects. Term *m*_*i*_ is the rumen microbial effect for the i^th^ animal ∼ NID(0,**M**σ^2^_m_), where σ^2^_m_ is the rumen microbial variance and **M** is the microbial relationship matrix, described in (2), i = 750 animals.

#### Association between rumen bacterial and archaeal OTU’s and host methane emission

The association between the relative abundance of each bacterial and archaeal OTU abundance with host methane production was conducted using linear mixed model analyses as proposed by Yu et al.[20], with the exception that OTU effects were estimated in place of allele substitution effects for genetic variants, as performed in genetic association analysis. The significance threshold was calculated using a Benjamini Hochberg false discovery rate correction for multiple testing. There were 189 archaeal and 3894 bacterial OTUs tested, and the microbiome wide significant threshold at FDR of 15% was in -log_10_(*P*) scale 2.17.

#### Microbial community analysis

A principal coordinate analysis (PoCA) was conducted to investigate similarities or dissimilarities using a distance matrix from the archaeal and bacterial rumen community composition. The Bray-Curtis coefficient was employed separately for the archaeal and bacterial OTU tables to create sample-summary matrices, which were further explored using non-metric multidimensional scaling (NMDS)[21]. The effects of environmental and genetic parameter effects on community structures were evaluated using the following model:

$$y_{ijkl}=\mu +sb_{j}+p_{k}+b_{1}\left(dim_{l}\right)+b_{2}\left(e^{‑0.065\;x\;diml}\right)+a_{i}+e_{ijkl}$$ (3)

where y_*ijklm*_ is the observed phenotype, e.g. PCoA 1 and PCoA2 for bacteria or archaea; *μ* is the model intercept; sb_j_ is the sequencing batch run fixed effect nested within the sequencing platform (j = 11 levels); *p*_*k*_, *b*_*1*_, *dim*_*l*_, *b*_*2*_, *a*_*i*_, and e_*ijkl*_ are as described in Eq (1). Additive genetic effects of host could not be detected from the bacterial and archaeal community structures for PCoA 2 and only herd environmental effects were significant. The distribution of *a priori* defined high and low emitters along PCoA1 was tested for bacterial and archaeal community structures, respectively by means of Mann-Whitney tests.

### Ethics statement

All handling of animals were conducted according to 'Metagenomics in Dairy Cows' protocol. The protocol and study were approved by The Animal Experiments Inspectorate, Danish Veterinary and Food Administration, Ministry of Environment and Food of Denmark (Approval number 2016-15-0201-00959).

## Supporting information

S1 Fig Methane (CH_4_) (g/day) measurements from 750 Holstein cows sorted by phenotypes corrected for environmental effects. The 10% highest CH_4_ emitters (red), 10% lowest CH_4_ emitters (green), and medium CH_4_ emitters (grey). *P*-value indicates significant differences between high and low CH_4_ emitters.
(TIFF)

S2 Fig Methane (CH_4_) (g/day) measurements from 750 Holstein cows against Energy Corrected Milk yield (ECM) (kg). The 10% highest CH_4_ emitters (red), 10% lowest CH_4_ emitters (green), and medium CH_4_ emitters (grey).
(TIFF)

S3 Fig Methane (CH_4_) (g/day) measurements from 750 Holstein cows against Body Weight (kg). The 10% highest CH_4_ emitters (red), 10% lowest CH_4_ emitters (green), and medium CH_4_ emitters (grey).
(TIFF)

S1 Table Heritability estimates and effect of abundance of rumen archaea and bacteria at taxonomic levels level on Methane (CH4 g/day). (XLSX)

S2 Table Bacterial OTU table of counts with prevalence in more than 50% of samples (Bac_OTU). (TXT)

S3 Table Archaeal OTU table of counts with prevalence in more than 50% of samples (Arc_OTU). (TXT)

S4 Table Individual cow phenotypes and metadata. (TXT)
