# Supplementary figures and images for: Host genetics and the rumen microbiome jointly associate with methane emissions in dairy cows
Source: PLoS Genet. 2018 Oct 12;14(10):e1007580. doi: 10.1371/journal.pgen.1007580 (PMC6200390; doi:10.1371/journal.pgen.1007580)

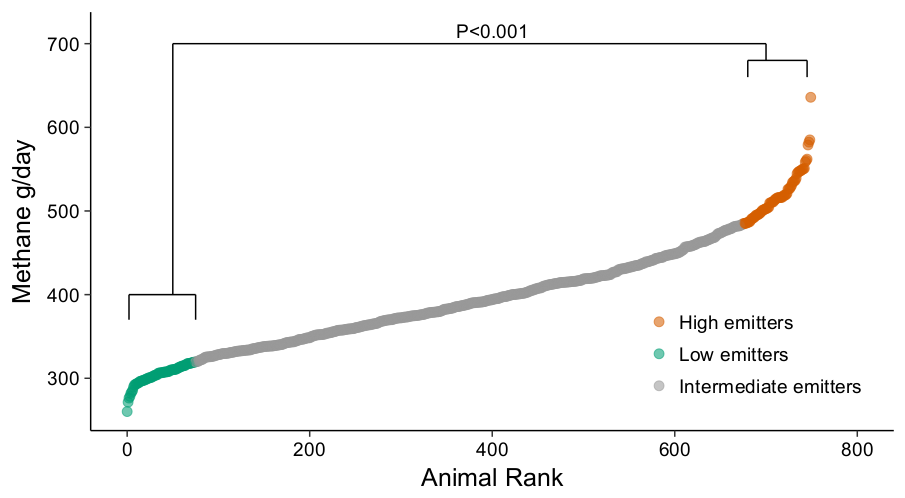

Supplement: S1 Fig — The 10% highest CH4 emitters (red), 10% lowest CH4 emitters (green), and medium CH4 emitters (grey). P-value indicates significant differences between high and low CH4 emitters. (TIFF) [file pgen.1007580.s001.tiff]

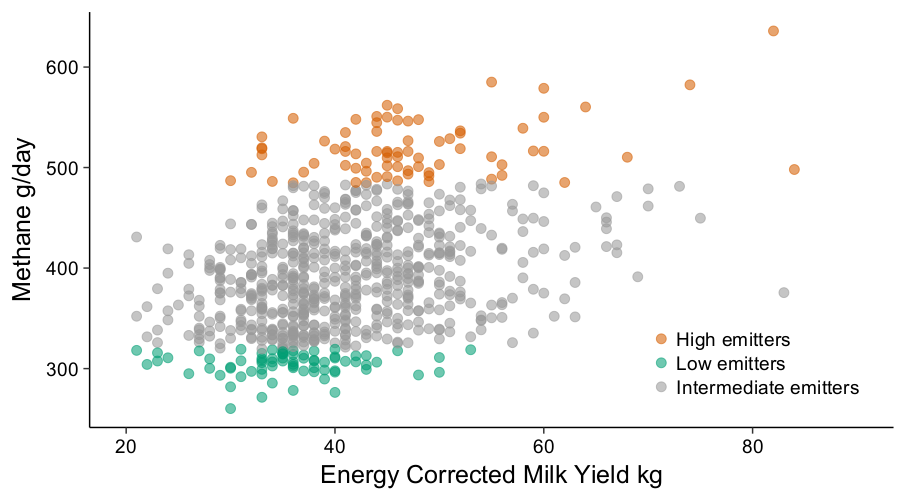

Supplement: S2 Fig — The 10% highest CH4 emitters (red), 10% lowest CH4 emitters (green), and medium CH4 emitters (grey). (TIFF) [file pgen.1007580.s002.tiff]

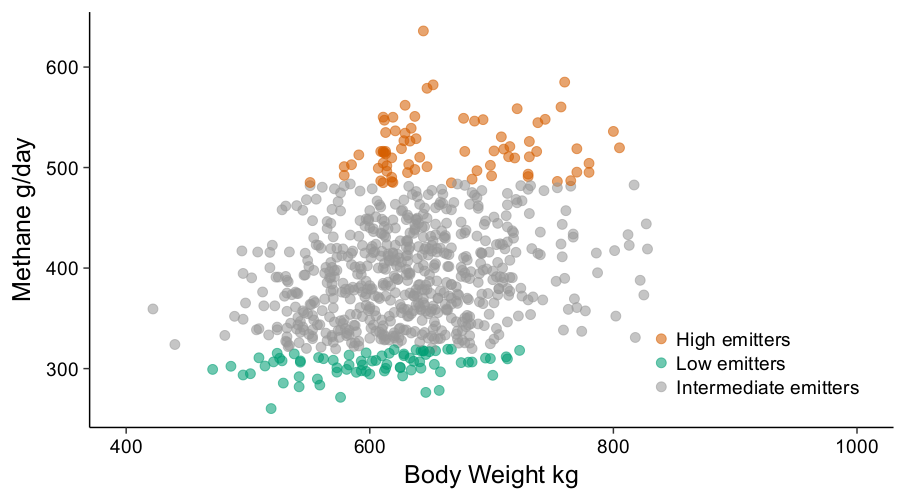

Supplement: S3 Fig — The 10% highest CH4 emitters (red), 10% lowest CH4 emitters (green), and medium CH4 emitters (grey). (TIFF) [file pgen.1007580.s003.tiff]
